# Village health worker motivation for better performance in a maternal and child health programme in Nigeria: A realist evaluation

**DOI:** 10.1177/13558196211055323

**Published:** 2022-01-28

**Authors:** Chinyere Mbachu, Enyi Etiaba, Bassey Ebenso, Udochukwu Ogu, Obinna Onwujekwe, Benjamin Uzochukwu, Ana Manzano, Tolib Mirzoev

**Affiliations:** 1 Health Policy Research Group, College of Medicine, 108000 University of Nigeria, Enugu, Nigeria; 2 Department of Community Medicine, College of Medicine, 108000 University of Nigeria, Enugu, Nigeria; 3 Department of Health Administration and Management, College of Medicine, 108000 University of Nigeria, Enugu, Nigeria; 4 School of Sociology and Social Policy, 150514 University of Leeds, Leeds, UK; 5 Nuffield Centre for International Health and Development, 150514 University of Leeds, Leeds, UK; 6 Faculty of Public Health and Policy, 150514 London School of Hygiene & Tropical Medicine, London, UK

**Keywords:** motivation, community health worker, realist evaluation

## Abstract

**Background:**

Community health workers play an important role in linking communities with formal health service providers, thereby improving access to and utilization of health care. A novel cadre of community health workers known as village health workers (VHWs) were recruited to create demand for maternal health services in the Nigerian Subsidy Reinvestment Programme (SURE-P/MCH). In this study, we investigated the role of contextual factors and underlying mechanisms motivating VHWs.

**Methods:**

We used realist evaluation to understand the impact of a multi-intervention maternal and child health programme on VHW motivation using Anambra State as a case study. Initial working theories and logic maps were developed through literature review and stakeholder engagement; programme theories were developed and tested using focus group discussions and in-depth interviews with various stakeholder groups. Interview transcripts were analysed through an integrated approach of Context, Mechanism and Outcomes (CMO) categorisation and connecting, and matching of patterns of CMO configurations. Motivation theories were used to explain factors that influence VHW motivation. Explanatory configurations are reported in line with RAMESES reporting standards.

**Results:**

The performance of VHWs in the SURE-P maternal and child health programme was linked to four main mechanisms of motivation: feelings of confidence, sense of identity or feeling of acceptance, feeling of happiness and hopefulness/expectation of valued outcome. These mechanisms were triggered by interactions of programme-specific contexts and resources such as training and supervision of VHWs by skilled health workers, provision of first aid kits and uniforms, and payments of a monthly stipend. The monthly payment was considered to be the most important motivational factor by VHWs. VHWs used a combination of innovative approaches to create demand for maternity services among pregnant women, and their performance was influenced by health system factors such as organisational capacity and culture, and societal factors such as relationship with the community and community support.

**Conclusion:**

This paper highlights important contextual factors and mechanisms for VHW motivation that can be applied to other interventions that seek to strengthen community engagement and demand creation in primary health care. Future research on how to sustain VHW motivation is also required.

## Introduction

Community health workers (CHWs) are recognised as a promising component of integrated health systems and an important part of the frontline primary health care (PHC) team.^1^ At times also referred to as lay health advisors, village health workers, community health aides and health extension workers, CHW provide basic public health services and health care including educating community members about health risks, promoting healthy behaviours or linking community members with providers at formal health care facilities. CHWs bridge community and formal health services, thereby increasing communities’ access to services, especially for those living in rural or underserved areas.^2^ Because CHWs reach community members at relatively low cost, they have been proposed and deployed to achieve a wide range of disease prevention and health system strengthening objectives,^3^ including in areas as diverse as maternal and child health (MCH), family planning, malaria control and environmental health.

As task-shifting is becoming more widely implemented, CHWs have an increasing number of tasks added to their list of responsibilities.^4^ Education, training, scope of work and employment status of CHWs vary across countries and health programmes but a common characteristic is that CHWs often lack a professional health care certification.^5^ CHWs can range from volunteers working without material compensation to paid employees, with other compensation mechanisms including periodic training stipends, financial incentives or preferential access to health care or microcredit.^5^ Irrespective of the potential contribution of CHW to advance universal health coverage, little is known about the factors determining their motivation in low- and middle-income countries (LMICs).^6^

CHWs play a central role in Nigeria’s efforts to reduce maternal mortality. The Subsidy Reinvestment and Empowerment Programme (SURE-P), launched in 2012, aimed to improve the lives of vulnerable populations using funds accrued from removal of petroleum subsidies.^7^ The maternal and child health (MCH) component (SURE-P/MCH) included the recruitment, training and deployment of midwives and a new cadre of CHWs known as village health workers (VHWs) to upgraded PHC centres, along with conditional cash transfers (CCTs) for mothers completing a series of MCH services including four antenatal care (ANC) visits, delivery in a PHC centre and postnatal care visit at 6 weeks^8^ VHWs were selected from their local communities, trained, supervised and paid a monthly stipend of about US$60 per month to mobilise pregnant women, promote ANC, encourage facility delivery by a skilled birth attendant and promote postnatal care for mothers and babies (family planning and immunisation). VHWs were also trained to provide a limited set of preventive health services such as birth preparedness counselling, health education, first aid for minor cuts and injuries, sanitation and hygiene education.^9^ VHWs had to be aged over 20 years, have a minimum of primary school education, able to read and write in English (where obtainable), resident in the community (preferably married), willing to serve in the community for at least 2 years and, for the northern parts of the country, be female as they were more likely to be acceptable in this Islam-dominated region.^9^ VHWs received an initial 1 week training and bi-annual refresher trainings. Prior to the commencement of VHWs activities, strong multi-level advocacy and sensitization were carried out at all levels, including in the communities.^9^

The SURE-P programme’s deployment of VHWs aligns with the global drive to use lower cadres of skilled and unskilled workers to optimize access to and efficiency of universal health care provision and accelerate achievement of the SDGs.^10^ Understanding the factors that motivate CHWs to perform their roles is as important to sustainability and this study seeks to contribute to this knowledge by examining the contextual factors and the mechanisms through which CHWs are motivated to perform their roles in the SURE-P/MHC programme.

## Methods

The study formed part of a larger project that evaluated the impacts of the SURE-P/MCH programme using realist evaluation,^11^ a theory-driven evaluation approach that builds, tests, validates and refines theories, with a specific focus on VHW motivation. Realist evaluation provides a means to understand the resources or opportunities presented by a given intervention that enable actors (e.g. policymakers, implementers and service users) to make it work.^12^ The projected effectiveness of an intervention is described in Context-Mechanism-Outcome (C-M-O) configurations, including explanation(s) of (i) why intervention outcomes turned out as they did and (ii) how the intervention/s responded to underlying mechanisms and in what contexts.^13^

### Theoretical framework

We drew on Herzberg’s two-factor theory and Vroom’s expectancy theory to understand how VHWs were motivated to carry out their duties, and the (group of) factors acting at macro, meso and micro levels that influenced their motivation. Herzberg’s two-factor theory considers motivational factors that lead to job ‘satisfaction’ (e.g. educational opportunities, sense of achievement, intrinsic interest in the work and involvement in decision-making) and hygiene factors that cause job ‘dissatisfaction’ when they are absent (e.g. salary, good working conditions, recruitment policies and administrative practices).^14, 15^ Motivational factors can be intrinsic or extrinsic to the individual whereas factors linked to job dissatisfaction (i.e. hygiene factors) are contextual and extrinsic to the individual. The theory stipulates that improving motivational factors increases job satisfaction while of hygiene factors decrease job dissatisfaction. Vroom’s expectancy theory is a process theory which focuses on outcomes, defined as an action-outcome estimate. People choose their behaviours (effort level) based on their perceptions of whether the behaviour is likely to lead to valued outcomes.^16^ Vroom introduced the concepts of expectancy (increased effort will lead to increased results), instrumentality (if you perform well, you will receive a valued outcome) and valence (value placed on the expected outcome).

### Study design

The evaluation was carried out in Anambra State, one of the 36 states in Nigeria, with a population of about 4.1 million and a mix of urban and rural areas. Maternal and child health services are primarily accessed from PHC facilities, each of which covering a given catchment population. The SURE-P intervention was first implemented in 12 PHC facilities, with another 12 facilities included in a second phase of the intervention. In this paper, we only report on first phase health facilities as they had a longer experience of the programme. The realist evaluation comprised three interrelated steps.^13^ We first developed an initial working theory based on a CMO configuration: *In a context where pregnant women are incentivised (free drugs, free ANC services, mama kits, CCT) to access MCH care and where VHWs are trained, paid a regular stipend and provided with resources (VHW kits) to enable them to sensitise and mobilise pregnant women and support them to access facilities (C), these VHWs will feel more recognised by communities and will be motivated to encourage and accompany pregnant women to facilities for MCH services (M). This will contribute towards increased and sustained utilisation of MCH services by the pregnant women (O).* In the second step, we used information from 16 in-depth interviews (IDIs) and 32 focus group discussions (FGDs) to build an initial programme theory for VHW motivation. Finally, the initial programme theory on VHWs was tested and refined alongside the CMO configurations developed in steps 1 and 2 above, using information from nine IDIs.

### Sampling and data collection

Focus group discussions were conducted with all VHWs in eight first phase facilities. FGDs were also conducted with service users, their family members and representatives of ward development committees (WDC) in eight first phase facilities.

In step 2, we additionally carried out IDIs with six health facility managers and ten health facility workers, followed by IDIs with nine VHWs in step 3 to allow for in-depth exploration of identified themes identified in step 2, in which all the VHWs participated. The IDIs focused on the factors that motivated the VHWs to carry out their duties, and how and in what combinations (if any) these factors worked to trigger mechanisms. We then used this information to develop a C-M-O template which consolidated patterns of explanations.

Study participants were provided with a study information sheet and those that expressed a willingness to participate were invited to a PHC centre or to the village hall, depending of their preference, for the interview. Interviews were conducted by three pairs of qualitative researchers who had received training in realist interviewing, with each lasting 45–60 minutes. Data collection tools for this study are presented in the Online supplement. Table 1 summarises the data collection methods across the study steps.

**Table 1.** Summary of the steps in the study and methods of data collection.

Steps	Method of data collection
1. Developing initial working theories (IWTs) of CMO configuration	Literature review Stakeholder consultations
2. Refining IWTs and building of initial thematic programme theory for VHW motivation with empirical data	FGDs (*n* = 32) Village health workers (FGDs) = 8 Service users (FGDs) = 8 Family members of service users (FGDs) = 8 Ward development committee (FGD) = 8 IDIs (*n* = 16) Facility managers (also health workers) (IDI) = 6 Other health workers (IDIs) = 10
3. Testing of programme theories with empirical data	IDIs (*n* = 9) Village health workers (IDIs) = 9

### Data analysis

Data were analysed using a realist reduction approach consisting of iterations of inductive and deductive analysis. The analysis was performed by four authors (CM, EE, BE and AM), one of whom is skilled and experienced in realist evaluation. Analysis was guided by our overarching theory which centres on the presence or absence of resources given (by the programme) and/or existing resources and how the VHWs interacted with these resources to produce behaviours which manifested in their actions.^17^ Varying explanatory configurations identified from the data are reported in line with the Realist And Meta‐narrative Evidence Syntheses Evolving Standards II (RAMESES).^16^

Interviews were transcribed verbatim and each transcript was read by two researchers to identify themes and sort them into context, mechanisms and outcomes. Identified themes were compared and synthesised across transcripts, using the CMO categories. This was followed by connecting and matching patterns of CMO configurations across transcripts to determine how the causal mechanisms played out and produced similar or distinct outcomes. Step 2 interviews were used to build and record initial relationships and linkages between contexts (resources), mechanisms and outcomes, and to generate proposed CMO configurations. Then, step 3 interviews were used to explore the proposed CMO configurations; that is, the effect of context, the proposed enabling mechanisms and the extent to which the outcomes had been achieved or not, and if not, why.

### Ethics approval

Ethical approval was granted by the School of Medicine Research Ethics Committee at the Faculty of Medicine and Health at the University of Leeds (ref: SoMREC/14/097) and the Health Research Ethics Committee at the University of Nigeria Teaching Hospital (ref: NHREC/05/02/2008B-FWA00002458-1RB00002323). Respondents were informed about the purpose of the study and their roles and rights as participants. Voluntary written consent was obtained from all the participants prior to the interviews.

## Results

**Figure 1.**
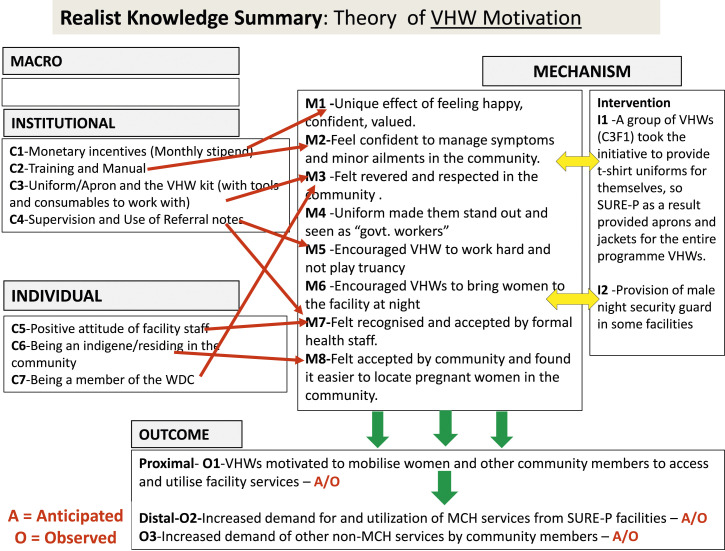
Realist knowledge summary of theory of village health workers motivation in the Subsidy Reinvestment Programme intervention in Nigeria.

Four distinct mechanisms of VHW motivation were identified: (i) feelings of confidence and expectation of good performance, (ii) sense of identity or feeling of acceptance, (iii) feeling of happiness and (iv) hopefulness/expectation of valued outcome. The following presents these mechanisms, with illustrative quotes from participants, as well as identified contextual enablers and constraints. Table 2 presents the CMO configurations, while Figure 1 illustrates the CMO templates for visualising the causal linkages of CMO configurations.

**Table 2.** CMO configurations of VHW motivation from realist interview findings.

CMOc 1: Feelings of confidence	In the SURE-P programme, VHWs were trained to be more knowledgeable, equipped with necessary materials (VHW kits) to provide first aid care and supervised by skilled health workers to ensure they adhered to guidelines (C/R). This made the VHWs feel confident in their capacity to mobilise pregnant women to utilise maternity services from PHCs (M). It also made them believe they were capable of mobilising pregnant women if they put in good effort (M). This resulted in better and innovative demand creation activities by VHWs, and an increased demand for antenatal and delivery services from PHCs by pregnant women (O)
CMOc 2: Sense of identity and belonging	Providing VHWs with a uniform (crested T-shirts or aprons), equipping them with necessary materials (VHW kits) to provide first aid for pregnant women and supervision by skilled health workers (C/R) gave VHWs a sense of identity and feeling of acceptance or belonging to the human resource for health (M). Their uniforms and kits earned them respect in the community. This resulted in better performance at mobilising pregnant women for maternity services offered in PHCs (O)
CMOc 3: Feeling of happiness	VHWs received a monthly stipend/salary (C/R) which made them feel happy (M) to do their work of mobilising pregnant women from the communities (O). VHWs transferred this happiness to their clients through some of the approaches they used to mobilise pregnant women for ANC. Their clients looked forward to visiting the PHCs and participating in the side attractions provided by VHWs (O)
CMOc 4: Hopefulness/expectations of valued outcomes	VHWs adopted some innovative approaches for mobilising pregnant women from their communities (C) because they believed (were hopeful) that such approaches would produce more results in terms of increase in demand for maternity services (M). This increased their commitment to mobilise pregnant women (O). The performance of VHWs was monitored using logbooks in which number of pregnant women mobilised was recorded by VHWs and validated by health facility staff, and there was an increase in the number of pregnant women mobilised (O)

Note: CMOc: Context, mechanisms and outcomes configurations; VHW: village health workers.

### Feelings of confidence

VHWs viewed the training and supervision that they had received during SURE-P in a very positive light. All VHWs mentioned that the trainings had equipped them with the knowledge they required to offer counselling and health education to pregnant women and caregivers of children under 5 years, and strengthened their skills in mobilising these women and their spouses, where necessary. Supervision, provided by health facility staff and the state programme coordinators, was perceived by many VHWs to have contributed to ensuring that they adhered to practice guidelines and standards which they were provided with during the training. Some VHWs also noted that the kits they were given equipped them with the necessary materials to provide first aid care to community members. The activities gave VHWs a feeling of confidence and empowerment to perform assigned roles of creating demand for maternal health among pregnant women in the communities.

In keeping with the expectancy variable of Vroom’s motivation theory, having been equipped with the right material resources, the right knowledge and skills to do the job, correct information about the job and supervisor’s support to get the job done, VHWs were confident that their efforts at mobilising pregnant women would lead to increased demand for maternity services from PHC centres. As a consequence, the VHWs used innovative approaches to create demand such as incorporating activities such as singing and dancing during mobilisation campaigns.

### Sense of identity, belonging and respect

VHWs were provided with uniforms (crested T-shirts and aprons) which distinguished them from the rest of the community and elevated them to a status of ‘health service provider’. Some VHWs, who had been engaged in delivering community-based health interventions in the past, did not have a uniform in their previous role and they did not feel part of the human resource for health. The crested SURE-P uniform made VHWs feel integrated into and accepted as a part of the health workforce. This feeling of acceptance or belonging was further strengthened by the supportive supervision the VHWs received from the formal and skilled health workers. Moreover, their apparel and association with PHC facility staff earned them the respect of the community:

We received some polo (T-shirt) from SURE-P and an apron too that dignified us such that we were addressed as ‘nurse’ (laughs). They gave us a kit which we used whenever we were on our way to give a talk…… When we kit-up we were respected in the communities. *(C2F2VHW_P1)*

This finding highlights that providing VHWs with a form of identification (e.g. a uniform) made them feel accepted by other health workers and gave them a sense of dignity that enabled them perform their roles in the community.

### Feeling of happiness and worthiness

The assurance of a monthly stipend was emphasised by all VHWs as an incentive that produced great motivation. They saw this financial reward as a confirmation that their work was viewed as valuable and this made them feel happy, worthy and willing to put in the effort. Some VHWs reported that the monthly stipend gave them some level of financial independence from their spouses, and increased their decision-making ability in the home. For instance, they would not always require spousal approval to purchase necessary food items in the home or seek health care that required payment of user fees. Some VHWs also reported that they were able to make future financial plans because the amount and timing of payment of stipend were predictable.

Whenever the alert [bank credit alert of monthly stipend] comes, it used to give me joy. My husband would even encourage me on the work because the money was of high importance to us. *(VHW_C2F4)*

It [monthly stipend] gave me the assurance that I am working. Because when you are being paid for the job you do, you will be confident doing the job, but when you are working without seeing the money, you will not be happy doing the work. But when the money is there, you will put in more effort doing the job because you are being paid and you are not working in vain. *(P2C2F1)*

### Hopefulness/expectations of valued outcomes

One of the components of VHW supervision during SURE-P was the use of logbooks in which clients’ data were recorded by VHWs, validated by health facility staff and monitored by a state-level supervisor who would provide commendations or criticisms in the logbook based on number of pregnant women mobilised. VHWs stated that this encouraged them to introduce and use various innovative approaches to mobilise pregnant women and as they implemented them and observed positive results from clients (in terms of increased demand for maternal health services), the VHWs’ commitment to work increased and more pregnant women were mobilised. This motivation mechanism also aligns with the instrumentality variable of Vroom’s motivation theory. In addition to the tangible incentives that would accompany good performance, VHWs were aware of intangible outcomes such as commendations/praise or criticisms.

We had a record that we document information concerning the number of people that we brought and at the end of the month the record is taken to the local government. So, it made us work extra hard and when we do bring people we get praised by the [facility manager]. She makes comments any month we persuaded lots of people and the comment would serve as encouragement to us. *(C2F2VHW_P2)*

### Contextual enablers and constraints

VHWs created demand for services among pregnant women, and continued awareness in the community about free or subsidised maternal health services. They used a combination of approaches including repeated visits at various sites, involvement of male spouses, provision of side attractions during ANC visits, accompaniment of clients during ANC and delivery visits and advocating for clients’ rights. Their work was enabled or constrained by a range of meso-level contextual factors within and outside of the organisation, as well as interactions between VHW incentives and other components of the SURE-P maternal and child health intervention.

Meso-level contexts within the organisation included organisational capacity; organisational culture of team work, task sharing and collegial support for colleagues; availability of skilled health facility managers and health workers; and attitude of PHC staff to VHW clients women referred by VHWs.

*Organisational capacity.* Working with a team of skilled health workers enabled VHWs learn how to identify complications in pregnancy, and how to recognise other health problems.

*Team work and collegial support* ***.*** Community outreach was jointly planned and implemented by VHWs and PHC staff. Some VHWs worked with CHEWs in the health facility and the community. This enabled task sharing and support for colleagues, with facility managers reported to be accommodating and approachable:

We were divided into four, we had two sets, some went to two villages and we went to the other two villages, that was how we covered the town then… It [relationship with colleagues] helped me because when we were working with them, … It [referring to the support VHW received from PHC worker] made us to be friendlier. In fact, we were working as mother and child, we worked well with them. I still have good relationship with them up till now. *(P2C1F1)*

*Attitude of health facility staff.* Facility managers and other health facility staff were receptive and considerate of clients. They took good care of clients and this made the work of mobilising and referral easier for VHWs.

Meso-level contexts outside the organisation refers to the relationship of VHWs to members of the community; community acceptance of VHWs; availability of resources such as security; means of transportation to remote parts of the community.

*Citizenship and residency status in the community*. Being a native of the village, or a member of religious and kindred groups facilitated contact with people for VHWs. Not being a permanent resident of the community could hinder access to people’s homes for another VHW, but this was offset by her being a native of the village**.**

It [being a resident of the community] helped me because if I am a visitor, I wouldn’t know the places well, so being an XX person helped me to know the places well. I know all the places in XX, then the person working in YY knows about YY, that was how they picked one person each from the six villages we have. *(P2C2F1)*

*Community acceptance of VHWs and community support.* Communities recommended trusted members to be selected as VHWs and because VHWs were selected by their communities, they received the cooperation of community members and were able to gain access to their homes to mobilise pregnant women to utilise maternity services in PHCs.

We the WDC [ward development committee] we’re the ones that nominated them and we know their capability before nominating them. They are hard working. They go into the villages and motivate pregnant women to come to the health centres. *(C1F1WDC_P5)*

Interactions with other components of SURE-P MCH interventions included resources that enabled VHWs to encourage pregnant women to utilise available maternity services.

*Availability of midwives and CHEWs round the clock*. VHWs found it easier to encourage their clients to deliver in a PHC if there was assurance that a skilled health worker would be available at all times. It was seen to be particularly important to clients that a midwife or senior CHEW was present to monitor and deliver their babies if they went into labour at night. SURE-P ensured that an adequate number of skilled health workers was present so allowing pregnant women to be confident to plan for PHC-based delivery.

*Provision of free mama kit to pregnant women* ***.*** The mama kit was freely given to all pregnant women that attended ANC in SURE-P health facilities and contained essential materials for delivery and new born care, including sanitary pads, receiving blanket, sanitary cord clips, antiseptic solution and cotton wool. These materials are usually purchased by pregnant women and add to the cost of delivery. The anticipation of receiving a free mama kit encouraged pregnant women to seek maternity care at the PHCs.

*Free ANC services and subsidised price of delivery* ***.*** Similarly, as antenatal care services were free of charge and delivery was highly subsidised, it was easier for VHW to encourage women to use these services. Also, women were less dependent on their spouses in deciding where to deliver. VHWs also found it easier to persuade men to permit their wives to utilise maternity services from PHCs because of the free or subsidised cost.

*Provision of conditional cash transfers (CCTs) to pregnant women* ***.*** Some pregnant women in selected PHCs received CCTs to utilise maternity services which also eased the work of VHWs in benefitting communities. Conversely, where CCTs were not provided, VHWs found it more difficult to encourage pregnant women to use PHCs. This was particularly the case where women were aware of CCTs being available in another community.

## Discussion

**Figure 2.**
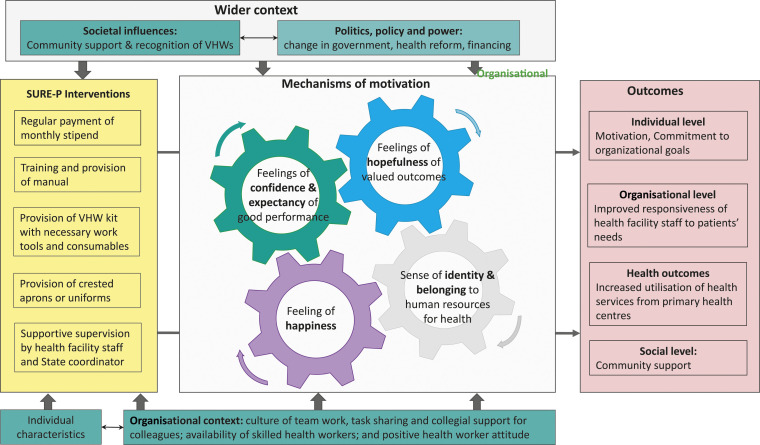
Theoretical framework of village health workers motivation (adapted from Ebenso et al., 2020).^28^

This study provides important insights into how and under what circumstances village health workers were motivated to mobilise pregnant women to utilise maternity services offered in PHC centres in the context of implementing a maternal and child health programme in Nigeria. Our observation informed the development of a theoretical framework of CHW motivation, which is illustrated in Figure 2.

VHWs in this study were motivated by a range of factors that we broadly refer to as incentives, including training, supervision, materials, crested aprons and a monthly stipend, as well as the outcome of ‘innovative’ approaches they used to mobilise women from communities. The interplay of these factors increased their confidence in their ability to mobilise pregnant women, giving them a sense of identity and belonging, and making them feel happy.

Monetary incentives in the form of monthly stipends were seen as the greatest motivation to work, creating a sense of achievement and a valued source of (additional) income on which they could depend on to support their households. Several studies on CHW motivation in LMICs highlight that monetary incentives are necessary, and our study supports other work showing that CHWs value the financial independence (and decision power) they gain from receiving financial compensation.^18-20^ Regularity and pattern of payment were important contributors to CHWs’ decisions to stay or leave the job; the ability to make financial plans was particularly valued. Other work has demonstrated that lack of financial compensation for CHWs led to attrition of CHWs, poor demand for MCH services and a reversal of gains in MCH outcomes that are attributable to the work of CHWs.^19, 21^

Training and supervision are recognised motivating factors for CHWs because they facilitate learning, contribute to improvement in knowledge and skills, and boost confidence.^18, 22^ In this study, VHWs developed a sense of identity and feeling of acceptance from having a dedicated uniform, a VHW kit to enable the provision of first aid, and supervision by skilled health workers. The importance CHWs attach to being identified and recognised or accepted in the community as part of health workforce is widely documented in literature.^23-25^ CHWs desire to contribute to health improvements in their communities and require related materials to support them in their work, and being able to distinguish them from lay community members also serves to reinforce CHW’s roles in community health programmes.^19, 23-25^ The interplay between motivational factors of confidence, identity and acceptance suggest that these mechanisms are mutually reinforcing in improving performance of CHWs.

We additionally found that material incentives and supervision by skilled health workers made the VHWs feel accepted by formal health workers. Acceptance of the value, talents or capabilities and performance of CHWs by the health system is a motivating factor for CHWs,^26^ and receiving the support and encouragement of peers can be seen as a distinct form of recognition, in addition to other incentives.^19, 26, 27^ Some CHWs join community health programmes because they seek the social prestige associated with the health profession,^18, 24^ and providing CHW with the appropriate tools increases their credibility and value in the community, which could be a source to increase motivation.^18, 20^

VHWs in our study used a range of innovative approaches to mobilise pregnant women and so increase demand for formal maternity service. This finding aligns with Vroom’s expectancy theory that links a person’s motivation to their efforts and performance. VHWs were provided with the right resources and skills to mobilise pregnant women, and, with supervision and support from formal health workers, were led to belief in their ability to perform well, and that if they did a good job, they would receive commendation and praise from their supervisors. This could explain their decision to try novel approaches to mobilise pregnant women in their communities.

We identified a range of additional factors that interacted with the motivating factors described above to enhance or, in the case of CCTs, make it more challenging for VHWs to perform their tasks. Enabling factors included the availability of skilled health workers in SURE-P, along with material resources (mama kits and CCTs) and free and subsidised maternity services. This highlights that VHWs require a supportive environment to perform well. Our study supports other work that has highlighted the role of organisational capacity, culture and policies for health worker motivation,^28^ and we have identified the importance of teamwork, task sharing and collegial support in this context. Our findings also support work that has found that CHWs are motivated when their peers and supervisors are supportive and encouraging,^20^ and that skilled health facility managers and health workers create opportunities for CHWs to acquire new knowledge and skills.^20^

Since CHWs interface between the community and the health system, the attitude of health facility staff to clients and the relationship between CHWs and the community affect the performance of CHWs.^19, 24^ In the SURE-P MCH intervention, VHWs recognised the need for their clients to be treated with dignity and respect by health facility staff, and they often accompanied clients to PHCs to ensure this was done. We found that a positive attitude of PHC staff contributed to improving the performance of VHWs and their motivation to refer clients to the PHC. We further show that VHWs’ residency status in the community and membership of community groups further enhanced their performance, along with community acceptance and cooperation, which was guaranteed since all VHWs were recommended by their communities. Community acceptance, trust and support are critical for the success of community health worker programmes.

With the withdrawal of funding for the SURE-P intervention, policymakers and programme managers must derive other means to sustain payment of CHWs, or else it will be difficult to retain them in primary health care. Future studies could apply choice experiments to explore the combinations of monetary and non-monetary incentives that are preferred by VHWs. Economic evaluation studies could be used to also determine the most cost-effective combinations of monetary and non-monetary incentives that can produce the expected performance.

### Limitations

In this retrospective realist evaluation study, researchers contended with social desirability bias. In anticipation that the SURE-P MCH programme could be reactivated and VHWs reinstated, some respondents appeared to share information that they believed the research team expected to hear. The effect of this was reduced by triangulation of data sources, namely by analysing data from VHWs, clients and health facility managers. With respect to external validity, although we achieved urban-rural geographic coverage, our theory was only tested in one state and this must be considered when interpreting our findings; further work is required to understand the applicability of our findings to other settings.

## Conclusions

This paper contributes knowledge on motivational factors for CHWs, and it provides new insight into the patterns of motivational mechanisms of salaried CHWs. We found that CHWs are motivated by various combinations of monetary and non-monetary incentives, and that they particularly value the monetary incentives. Monetary compensation is necessary for the sustainability of CHW programmes. CHWs’ performance was further influenced by health system factors such as organisational capacity and culture, and societal factors such as relationship with the community and community support.
